# Calcium sources can increase mechanical properties of 3D printed bioactive hybrid bone scaffolds

**DOI:** 10.1039/d3ra07946e

**Published:** 2024-11-27

**Authors:** Agathe Heyraud, Francesca Tallia, Steven Chen, Jingwen Liu, Jishizhan Chen, Joel Turner, Gavin Jell, Peter D. Lee, Julian R. Jones

**Affiliations:** a Department of Materials, Imperial College London London UK julian.r.jones@imperial.ac.uk; b Faculty of Engineering Science, Department of Mechanical Engineering, University College London UK; c Division of Surgery and Interventional Sciences, University College London UK

## Abstract

Inorganic/organic hybrid biomaterials have been developed to obtain synergy of the inorganic and organic co-networks for implant and 3D printed scaffold applications, providing combinations of bioactivity, toughness and controlled biodegradation. SiO_2_–CaO_CME_/PTHF/PCL-diCOOH sol–gel hybrids previously showed potential for osteogenesis due to the addition of calcium to the silicate network of the hybrid, using calcium methoxyethoxide (CME) as the calcium source. Here, we investigate other calcium sources to improve mechanical properties and printability of the hybrid inks. The aim was to produce porous scaffolds with mechanical properties similar to trabecular bone. The original Ca-free hybrid composition SiO_2_/PTHF/PCL-diCOOH was highly elastic and the addition of Ca increased strength while introducing bioactivity, with hydroxyapatite formation in simulated body fluid (SBF), and no negative effects on the metabolic activity of human bone marrow stromal cells (hBMSCs). However, when the hybrid was 3D printed by Direct Ink Writing, the mechanical properties were insufficient for a load sharing bone scaffold. Alternative calcium sources were investigated here, using concentrated CME (cCME), calcium hydroxide (CH), calcium ethoxide (CE), and calcium ethoxyethoxide (CEE). CEE improved the overall printability and final structure of the hybrid scaffold obtained and apatite formed on its surface in SBF. This hybrid reached the highest stress at failure (0.55 ± 0.08 MPa) and toughness modulus (0.13 ± 0.03 MPa), with a corresponding strain of >50%. With this calcium source and the optimal 70 : 30 TEOS : CEE molar ratio, scaffold properties were optimised by increasing the strut size whilst maintaining the interconnected channel size >400 μm and increasing the inorganic : organic ratio. Using a TEOS : PCL-diCOOH ratio of 85 : 15 wt%, giving a final inorganic content of 35.7 wt%, showed the optimal mechanical properties with a stress at failure of 3.1 ± 0.54 MPa for strain of 26%, and a toughness modulus of 0.58 ± 0.06 MPa, whilst keeping an open porosity >38%. Compressive strength was within the lower range of trabecular bone (2–12 MPa), and there was no observed cytotoxic effect on hBMSCs, indicating potential for use of this hybrid for bone regeneration.

## Abstract 2

A bioactive hybrid material for bone implants, synthesised *via* the sol–gel route and incorporating calcium, and 3D-printed into an interconnected porous scaffold.

## Graphical abstract

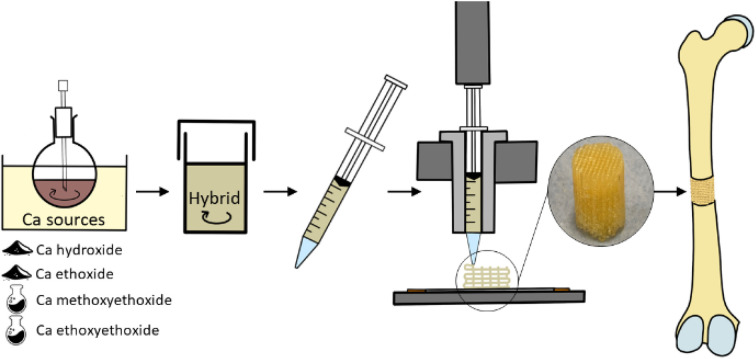

## Introduction

Hybrid biomaterials have interpenetrating organic and inorganic networks, often with a mix of covalent and dynamic bonds between the networks.^1^ The aim of using hybrids over conventional composites (*e.g.* inorganic particles in a polymer matrix) is synergy of their adaptable mechanical properties, ability to withstand cyclic load, and controlled biodegradation. Being able to alter the chemical and mechanical properties of the hybrids allows them to be optimised to match the properties of host tissues.^2^ In a hybrid, the organic and inorganic phases are indistinguishable above the nanoscale,^1^ enabling degradation as a single-phase material and cells to interact with both networks simultaneously.

In bone regeneration, bioactive ceramics and glasses are used as synthetic bone grafts, as they can bond with the bone and stimulate new bone growth.^1^ However, they are brittle and cannot bear cyclic loads. There is an unmet clinical need for bioactive scaffolds that can regenerate large bone defects, as on average 1 in 10 long bone fractures do not heal, even after treatment (non-union).^3,4^ Although conventional composites are available as synthetic bone grafts, they tend to be mixtures of demineralised bone matrix (collagen I) with a bioactive particulate, or granules in a putty, with no mechanical strength.^1^ Hydroxyapatite bioceramics have been 3D printed and showed good osteoinductivity with potential for maxillofacial bone regeneration, however not suitable for load bearing non-union repairs.^5^ Printable composites have been investigated, such as GelMA hydroxyapatite composites, showing good printability, osteogenic activity, however, mechanical properties did not match the desired trabecular bone.^6^ Load bearing composites have not found clinical success; the bioactive component can be masked by the polymer matrix, preventing bone bonding, and the polymer and inorganic components have differing rates of degradation. Combining organic polymers and inorganic bioactive glasses at the nanoscale enables simultaneous exposure of both networks to biological fluids and controlled degradation.^7^

Hybrids have been synthesised *via* the sol–gel route by hydrolysis of silicate based alkoxides such as tetraethylothosilicate (TEOS), incorporating a polymer into the process prior to the gelation of the sol, which formed a silicate network. Promising mechanical properties were attained in the following systems: SiO_2_/PTHF/PCL-diCOOH (compressive strength of 3 MPa at a strain of 28%, inorganic content of 25%, bulk),^8^ silica/gelatin (compressive strength of 0.05 MPa at 9% strain, inorganic content 40%, foam scaffolds),^9^ silica/poly(γ-glutamic acid) (compressive strength of 64 MPa estimated from elastic modulus of 2.0 GPa, inorganic content of 53%, bulk)^10^ and silica/methacrylate (compressive strength of 300 MPa at 14.2% strain, 50% inorganic, bulk).^11^ Some of the systems were also successfully 3D printed, by Direct Ink Writing, showing excellent resistance to cyclic loads: 1.2 MPa at 36% ^8^ (SiO_2_/PTHF/PCL-diCOOH), 1.0 MPa at 10% ^12^ (silica/gelatin). In each of these hybrids, the inorganic network was only silica. SiO_2_/PTHF/PCL-diCOOH, termed “Bouncy Bioglass” are particularly promising due a combination of covalent (*via* GPTMS coupling) and hydrogen bonds between the networks and their ability to self-heal and recover from deformation.^8^

According to *in vitro* studies on the properties of bioactive glasses, their bone bonding ability is attributed to forming a hydroxycarbonate (HCA) layer on their surface on exposure to body fluids,^13^ and their osteogenic properties are due to sustained release of their dissolution products of calcium ions and soluble silica.^14–17^ For the hybrids to exhibit both modes of bioactivity, calcium must be incorporated into the silicate network. However, this is not trivial. Sol–gel derived bioactive glasses of composition 70 mol% SiO_2_–30 mol% CaO did show potential for bone regeneration, provoking primary human osteoblasts to produce mineralised bone matrix without osteogenic supplements.^18^ Analysis of the calcium incorporation during synthesis showed that conventional calcium source used for sol–gel glass (calcium nitrate), required a heat treatment higher than 450 °C to incorporate the calcium into the silicate network.^19^ When polymers are incorporated into the sol for hybrid synthesis, they cannot undergo heat treatments, thus alternative calcium sources are needed to allow calcium to enter the silica network at room temperature.^20^ Other calcium salts have been investigated, such as calcium chloride and calcium acetate,^21^ but they still do not incorporate and recrystallise on the surface during drying.^10,20^ A new source had to be used: calcium alkoxides.^22^

The first calcium alkoxide to be used was calcium methoxyethoxide (CME) and was proven to incorporate into the silica network at temperatures below 60 °C, by solid state MAS-NMR studies, where calcium salts did not.^21,23,24^ CME hydrolyses when added to the hydrolysed TEOS solution, incorporating calcium into the wet gel. As the silica cross-links during ageing and drying, it maintains calcium in its network.^21,23,24^ CME was successfully incorporated into a SiO_2_–CaO/poly(γ-glutamic acid) bulk hybrids,^25^ SiO_2_–CaO/polyethylene glycol bulk hybrids,^26^ and SiO_2_–CaO/PTHF/PCL-diCOOH bulk hybrids^27^ which all showed apatite formation in SBF after 3 days and promising cell response. SiO_2_–CaO/poly(γ-glutamic acid) hybrids and their dissolution extract was not toxic to hMSCs and showed cell growth over a 7 days period.^25^ SiO_2_–CaO/polyethylene glycol bulk hybrid extracts showed good MC3T3-E1 cell viability after 3 days, increasing compared to the control after 6 days.^26^ SiO_2_–CaO/PTHF/PCL-diCOOH hybrid dissolution products showed good cell viability (above 70%) when exposed to MC3T3-E1 preosteoblast cell lines, passing the ISO standard cytotoxicity test.^27^ Strength of SiO_2_–CaO/PTHF/PCL-diCOOH^27^ bulk samples increased from 3 MPa at a strain of 28% (no Ca) to 64 MPa at a strain of 56% due to the incorporation of calcium to a 60 : 40 TEOS : CME molar ratio.^27^ Scaffolds were produced by Direct Ink Writing to achieve 500 μm channels and 100 μm strut size.^28^ As CME content increased, compressive strength increased, with 30 mol% calcium (70S30C_CME_-CL) achieving the highest strength and toughness of 0.90 ± 0.23 MPa and 0.22 ± 0.04 MPa, respectively. The 70S30C_CME_-CL scaffolds showed stable properties when immersed in simulated body fluid (SBF) for 90 days, forming HCA within 3 days.^28^ Culture media conditioned with the hybrid did not cause cytotoxicity to hBMSCs over 24 h. However, CME decreased the gelation time of the hybrid sol, forming an unreliable structure with reduced mechanical properties due to poor strut bonding from reduced polymerisation. This highlights the need to investigate different calcium sources which would not compromise the polymerisation and final structure.

Calcium ethoxide (CE) was tested in silica-gelatin and silica/PCL hybrids,^29,30^ with inorganic composition of 76 mol% SiO_2_–24 mol% CaO, and ^29^Si solid-state NMR showed that the Ca was part of the silicate network,^30,31^ more than when Ca salts were used. Ca release occurred within the first hour in SBF, with the Ca and P concentration in SBF decreasing continuously from 1 h to 168 h of immersion: confirming apatite formation.^29^ Calcium 2-ethoxyethoxide (CEE) was stated to have a lower sensitivity towards hydrolysis and condensation than CME and CE.^32^ This instability induces rapid gelation in presence of water, even from ambient humidity.^32^ The ability of CEE to have a stronger stability with water could form more homogeneous hybrid networks and was used as a coating for titanium implants. Si–Ca-PEG hybrids were made using GPTMS as the coupling agent with 56 wt% TEOS, 24 wt% calcium 2-ethoxyethoxide, and 20 wt% PEG.^32^ The mechanism of successful calcium incorporation from calcium alkoxides was attributed to dissolution of the alkoxide causing a rise in pH,^31^ liberating Si–O^−^ bonds without anions such as Cl^−^ preventing formation of SiO–^+^Ca bonds. Therefore, it was logical to try calcium hydroxide, which has some advantages over calcium alkoxides, such as commercial availability and cost (commercial calcium alkoxides seem to have reproducibility issues). Bossard *et al.* compared Ca integration into silica/PCL hybrids between use of calcium hydroxide, CE, and calcium chloride.^31^ Calcium hydroxide and CE both incorporated Ca into the silica network, but more Ca incorporated when CE was used.^31^ None of these calcium sources have previously been tested in hybrid systems for load bearing applications, such as bone implants.

The aim of this work was to determine the optimal calcium alkoxide source for production of 3D printed SiO_2_–CaO/PTHF/PCL-diCOOH “Bouncy Bioglass”. This adaptability of this hybrid's composition and geometry gives it a unique ability to match host trabecular bone with an open interconnected pore network, supporting and promoting tissue ingrowth, whilst providing mechanical stability to the patient.

## Experimental

### Materials

All materials were obtained from Sigma-Aldrich (Dorset, UK) and VWR UK, unless specified otherwise.

### Calcium sources synthesis

Following the synthesis method established by Pickup *et al.*,^33^ the concentrated calcium methoxyethoxide (cCME) solution was made by adding 1.5 g of calcium pieces (<1 cm, 99%) reacted to 24 mL of anhydrous 2-methoxyethanol heated to 80 °C using a paraffin bath, under argon atmosphere for 24 h, with continuous stirring at 600 rpm. The original CME solution is synthesised with 1 g of calcium pieces in 24 mL of anhydrous 2-methoxyethanol.^33^ The opaque grey solution obtained was centrifuged for 20 minutes at 6000 rpm, with the extracted solution stored at RT under inert conditions. The achieved concentration was 1.5 mmol mL^−1^. Higher concentrations are achievable with the same method, however, ≥2 mmol mL^−1^ CME became too reactive when synthesising the hybrids.

To synthesise calcium ethoxide (CE) powder, 2 g of calcium was placed in a round bottom flask with absolute ethanol (<0.005% water) in excess, here 60 mL.^34,35^ The flask was purged with argon and kept under inert conditions using an argon balloon, keeping all outlets shut. The solution was stirred at 600 rpm for 12 h at 70 °C, after which an opaque white solution was obtained. This is the CE formed in the excess ethanol. After 12 h, an outlet and a continuous argon flow were introduced, keeping the reaction under inert conditions, but allowing the ethanol to slowly evaporate. This was monitored and stopped once all excess solvent had evaporated, taking up to 5 h. The white powder obtained was stored under argon at 4 °C.

Calcium ethoxyethoxide (CEE) was synthesised by heating up 48 mL of 2-ethoxyethanol (99%) to 80 °C using a paraffin bath with continuous stirring at 600 rpm. The entire reaction was done under argon. 2 g of calcium was introduced in the heated flask, which was then re-purged. The temperature was increased to 125 °C and left to react for 20 h.^36^ This resulted in an opaque grey solution, similar to CME. It was then centrifuged for 20 minutes at 6000 rpm, the transparent red supernatant was extracted and stored at RT under argon.

### Hybrid synthesis with different calcium sources

To synthesise the hybrids, TEOS and the calcium source were homogenised for 3 h at 800 rpm at a ratio of 60 : 40 mol%, later using 70 : 30 mol% when optimising the final hybrid properties.^28^ In parallel the organic solution was synthesised reacting PCL-diCOOH (1 mol), (3-glycidyloxypropyl) trimethoxysilane (GPTMS, 2 mol), and boron trifluoride diethyletherate (BF_3_.OEt_2_, 0.5 mol) in tetrahydrofuran for 1.5 h at 400 rpm. A 70 : 30 wt% TEOS : PCL ratio was used for 60S40C_Ca_-CL hybrids. Once both the organic and inorganic solutions fully reacted, the inorganic solution was then added dropwise to the organic solution and left to stir at room temperature for a further hour. After which, deionised water was added to hydrolyse the TEOS and GPTMS, followed by 2 M nitric acid. For all hybrids, a TEOS to DI water molar ratio of 1 : 2 mol% was used to prevent rapid gelation and calcium reacting with water before TEOS and GPTMS hydrolysis. 1/6 v/v of 2 M nitric acid with respect to water was added. The amount of THF used and the hybrid reaction time after the addition of water and acid varied for each hybrid and is summarised in Table 1. When powder calcium sources (CH and CE) were used, the PCL-diCOOH to THF ratio was decreased to 50 mg mL^−1^ to further dilute the hybrid, as no additional solvent was introduced. The increased polymer content, due to the increase in THF and subsequent PTHF, was offset by using a ratio of TEOS : PCL-diCOOH of 80 : 20 wt%.

**Table 1:** SiO_2_–CaO/PTHF/PCL-diCOOH hybrids synthesised with different calcium sources (cCME, concentrated calcium methoxyethoxide; CH, calcium hydroxide; CE, calcium ethoxide; CEE, calcium ethoxyethoxide) and ratios of PCL-diCOOH : THF and TEOS : PCL-diCOOH for synthesis and reaction time. All initially synthesised with a TEOS : Ca ratio of 60 : 40 mol%

Calcium source for hybrid synthesis	PCL-diCOOH : THF (mg mL^−1^)	TEOS : PCL-diCOOH ratio (wt%)	Final reaction time before pouring into syringes (min)
cCME	100	70 : 30	30
CH	50	80 : 20	10
CE	50	80 : 20	20–30
CEE	100	70 : 30	5

The same reactant quantities and methods were used to synthesise both hybrid ink and bulk samples, with the optimised hybrid sol being liquid enough to be poured in both moulds for bulk, and syringes for printing. In this work, bulk monoliths were used for analysis of the hybrid behaviour in wet environment. The sol was poured in a Teflon mould (7 mL for a 70 mm diameter mould) stored in an airtight container for 3 days of ageing at 40 °C, followed by gradual drying for 7 days. The resulting hybrid monoliths of 1 mm thickness were finally cut with 8 mm diameter hollow punches.

To compare printability of the hybrids, the scaffolds were printed using the following parameters: 10 mm s^−1^ printing speed, 0.20 mm *z*-spacing, 0.20 mm printing nozzle and a 1 mm road width strut separation. Printed scaffolds were aged at 40 °C for 3 days in airtight sealed PMP pots, followed by 7 days of drying by opening the pots by ¼ revolution every day.

### Characterisation of the hybrids

Scanning Electron Microscopy (SEM, JEOL 6010 LA) was used to evaluate the architecture of the hybrid scaffolds. Secondary electron mode was used, with a voltage of 20 kV, a working distance between 13 and 20 mm, and spot size between 40 and 60 μm. Carbon tape was used to fix the samples on aluminium holders before coating them in a 10–15 nm layer of gold using a EMITECH K575X Peltier cooled coater. The top and bottom surfaces and cross sections were imaged to investigate apatite formation on the hybrid monoliths. As for the printed scaffolds, top surfaces and cross sections were imaged to measure the strut and channel size.

The scaffolds were scanned using X-ray microtomography (μCT, Nikon XTH225 ST) at 70 kV and 140 μA, with a voxel size of 4.0 μm, to characterise the porosity percentage and interconnectivity of channels. The μCT images were reconstructed (Nikon's CT Pro 3D) and a sub volume of interest (VOI, 2600 × 2600 × 2600 μm) was defined for quantification. First a 3 × 3 × 3 median filter was applied to reduce noise,^37^ then manual thresholding was performed, selecting the mid-point between attenuation peaks using Avizo (version 2021). “Label analysis” in Avizo was utilised for the measurement of porosity and interconnectivity (percentage of pores connected with each other and the exterior). Channel sizes and the strut equivalent diameter of scaffolds were measured using the open-source image processing program ImageJ with the BoneJ plugin.^38^ Two types of thickness maps were made for the optimised 70S30C_cCME_-85S15CL hybrid scaffold: one illustrating pore size and the other strut equivalent diameter.^39^

Fourier Transform Infrared (FTIR) spectroscopy was used on all the hybrid compositions to verify the presence of the functional groups corresponding to the organic and inorganic components of the hybrid system with different calcium sources. Compositional changes upon immersion of the different hybrid monoliths in SBF were also analysed. A Thermo Scientific Nicolet iS10 FTIR equipped with Smart Golden Gate for Single-Reflection Diamond ATR Analysis, with OMNIC software was used. 64 scans were collected at a resolution of 4 cm^−1^ for a spectral range of 400–4000 cm^−1^. Solid samples were prepared by manual grinding into a fine powder.

Simultaneous Differential Scanning Calorimetry and Thermogravimetric analysis (DSC/TGA) were performed on Netzsch Jupiter STA 449C instrument with Proteus software to process the acquired data. The hybrid samples were manually ground into a fine powder. Between 10 and 15 mg of the powder was placed in a platinum crucible, using an empty platinum crucible as the reference. A heating rate of 10 °C min^−1^ over a temperature range of 20 to 800 °C under continuously flowing air was used. This technique was used to analyse the hybrids final I : O ratio and any changes after immersion in SBF.

Compression testing was performed on the 3D printed hybrid scaffolds to assess and compare maximum stress and strain when varying the calcium source. The scaffolds were cut to 5 × 5 × 5 mm^3^ cubes, and the load was applied perpendicular to the plane of deposition during printing. A Bose Electroforce Series III mounted with a 450 N load cell was used with the Wintest software to collect the data, with a displacement rate of 0.5 mm min^−1^. The engineering and true stress and strain at yield were calculated. The toughness modulus was calculated by measuring the area under the stress–strain curve using the Origin Software (OriginLab Corporation, USA). DMA (Dynamic Mechanical Analysis) was performed on the same scaffold dimensions with the Bose Electroforce Series III used in parallel with the Wintest DMA software. Strain ranges of 1–5%, 5–9% and 9–13% were used, individually calculated for each scaffold from their measured height, the data collected at frequencies of 0.1, 1 and 10 Hz. DMA was performed to analyse the viscoelastic behaviour of the scaffolds and compare the stiffness of hybrids synthesised with varying calcium sources.

X-ray diffraction (XRD) was carried out using a Bruker D2 PHASER desktop diffractometer, the data was analysed with a PANalytical X'Pert HighScore software. A CuKα tube anode was used (*λ* = 1.5418 Å) and the generator settings fixed at a voltage of 30 kV and current intensity of 10 mA. A nickel filter was used to remove Kβ. Each pattern was recorded in the range of 10 to 80°. The step size and time per step were set at 0.035° and 0.35 s per step, respectively. Hybrid samples were manually ground into a fine powder and flattened in a single crystal silicon sample holder, to prevent background noise. XRD was first used to understand if any pure form of the calcium source was still present in the hybrids, such as calcium hydroxide, and then to detect the formation of crystallised apatite on otherwise amorphous hybrids after immersion in SBF.

A Thermo Scientific ICAP 6300 Inductively coupled plasma-optical emission spectroscopy (ICP-OES) with autosampler was used with the iTEVA software to determine the concentration of Ca, Si and P in SBF solution after immersion of hybrid scaffolds. The aim was to monitor the controlled release of silica and calcium ions from the hybrid degradation and analyse potential apatite formation on the hybrids.^27^ Due to the high content of calcium in SBF itself, the samples were diluted by a factor of 10 with DI water (1 mL of the aqueous sample and 9 mL of DI water). The standard solutions for calibration were prepared with Si, Ca, and P at 0, 0.1, 0.2, 0.4, 0.8, 1, 5, 10 and 20 μg mL^−1^. For the dissolution studies, timepoints of 0–8 h, 1, 3, and 7 days were selected to analyse the effect of SBF on hybrids with different calcium sources. Samples were weighed to measure mass loss post dissolution. They were then rinsed three times in DI water prior to the immersion in SBF to ensure no reaction by-product were left over (such as BF_3_·OEt_2_) and placed in SBF using a 1.5 mg mL^−1^ concentration. This concentration is commonly used for testing apatite formation on bioactive glasses in SBF.^27,40^ The samples were then kept in an incubator at 37 °C and 120 rpm.^41^

### Cell culture

Human bone mesenchymal stem cells (hBMSCs) were purchased from ScienCell (UK) at passage 1. hBMSCs were cultured in mesenchymal stem cell media (MSCM – ScienCell UK) containing 5% (V/V) FBS, 1% (V/V) penicillin/streptomycin (P/S) and 1% (V/V) mesenchymal stem cell growth supplement, following manufacturer's instructions. For experiments assessing the cell viability with 70S30C_CEE_85S15CL hybrid dissolution products, hBMSCs were used between passage 3 and 5.

### 70S30C_CEE_85S15CL hybrid dissolution products

70S30C_CEE_85S15CL hybrid discs of 8 mm diameter and <1 mm thick were sterilised by 3 washing of DI water, 70 and 100% ethanol, and irradiated under UV light for 3 h then air dried for 24 h. The hybrid dissolution products (DPs) were made by immersing discs in non-supplemented MSCM (10 mg mL^−1^) and incubated on a roller shaker at 37 °C for 72 h. DP stocks were then filtered using a 0.2 μm PES syringe filter. The 10 mg mL^−1^ stock concentration was used for a dilution series of 25, 50, 75, and 100% in non-supplemented MSCM, followed by addition of FBS, P/S, and growth factors. Quantification of Si, Ca, and P in DPs was performed by ICP-OES in addition to pH measurements.

### Metabolic activity with 70S30C_CEE_85S15CL hybrid dissolution products

hBMSCs were seeded at 4000 cells per cm^2^ on tissue culture plastic and allowed to attach for 24 h before the addition of DPs. Prior to cell culture, hybrid DPs were pre-incubated at 37 °C and 5% CO_2_ for 4 h to decrease pH. The metabolic activity of hBMSCs with 0, 25, 50, 75% dilutions of 70S30C_CEE_85S15CL hybrid DPs (and a control containing only MSCM) were assessed after 24 h using Prestoblue dyes (Invitrogen, Merck). Briefly, Prestoblue solutions were prepared at a 1 : 10 dilution with supplemented MSCM warmed to 37 °C prior to addition to cells. Supernatants in wells were removed, replaced with Prestoblue dye and incubated at 37 °C with 5% CO_2_ for 1 h. Cell supernatants containing Prestoblue dye were then transferred to a black 96 well plate and fluorescence of each well measured at 560 : 660 nm (excitation : emission) using a Tecan M200 Pro photospectrometer (Tecan, UK).

## Results and discussion

### Hybrid network formation with varying calcium sources

Initially, SiO_2_–CaO/PTHF/PCL-diCOOH hybrids were made in bulk form with different Ca sources, with molar ratio of TEOS : Ca of 60 : 40 (termed 60S40C_Ca_-CL), to investigate efficacy of Ca incorporation from the newly synthesised calcium sources (cCME, CH, CE, and CEE), compared to the 60S40C_CME_-CL hybrids synthesised previously.^28^ Nominal inorganic/organic weight ratio was 25 : 75.

**Fig. 1:**
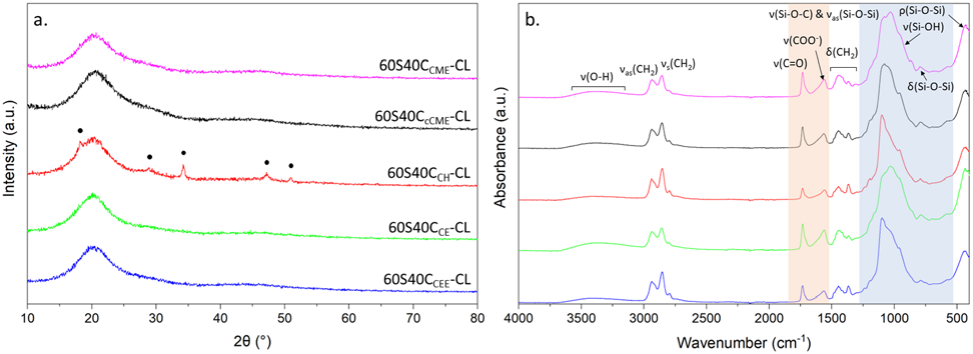
XRD and FTIR of SiO_2_–CaO/PTHF/PCL-diCOOH hybrids with different calcium sources, with molar ratio of TEOS : Ca of 60 : 40: (a) XRD patterns; annotated peaks match ICDD card number 01-070-5492 for Ca(OH)_2_; (b) FTIR spectra; blue region highlighting bands representative of the inorganic network, and the orange region the carbonyl and carboxylate bands from the interaction of calcium with PCL-diCOOH.

XRD was used to determine whether the hybrids were amorphous, and FTIR confirmed presence of the correct chemical bonds (Fig. 1). All hybrids made with calcium alkoxides were amorphous, according to XRD (Fig. 1a), but the XRD pattern of the 60S40C_CH_-CL hybrid shows peaks at 18°, 29°, 34°, 47° and 50° 2*θ*, matching ICDD card number 01-070-5492 for Ca(OH_2_), calcium hydroxide,^42,43^ meaning not all CH reacted with the hybrid network. For the hybrids made with calcium alkoxides, no calcium oxides or hydroxide peaks are visible: the calcium was integrated into the network and there was no excess. In the FTIR spectra (Fig. 1b), bands corresponding to the Si–O–Si asymmetric stretching (1000 and 1100 cm^−1^), bending (790 cm^−1^) and rocking (470 cm^−1^) and Si–O–C stretching were present for all hybrids, confirming formation of the silica network.^44^ Spectra of 60S40C_CEE_-CL and 60S40C_CH_-CL hybrids have less intense Si–O–Si bands, due to their faster gelation post hydrolysis. The carboxylate anion band, COO^−^, at 1580 cm^−1^, characteristic of excess calcium bonding to free terminal chain of the PCL-diCOOH^27^ is present for all hybrids, expected for this high calcium content (TEOS : calcium molar ratio of 60 : 40).^27,28^ The 1725 cm^−1^ band of the carbonyl group was less intense for the 60S40C_CH_-CL hybrid, showing its inorganic : organic (I : O) network may differ from the others.

Due to the similarity of the hybrids synthesised with CME and cCME, only concentrated CME was used as a comparison to the other calcium sources for the rest of this study. Table 1 summarised the PCL-diCOOH : THF and TEOS : PCL-diCOOH ratios used to synthesise the hybrids, to obtain the final I : O ratios of the hybrids, measured using TGA, Table 2. Hybrids made with CEE and cCME had I : O ratios similar to those expected. However, hybrids made with the powder calcium sources had I : O ratios lower than predicted: these required an increase in THF to prevent excess gelation. This was the best solution to prevent the addition of a new solvent to the system, and the calcium powders did not dissolve in ethanol. It likely caused an increase in PTHF which was compensated by increasing the TEOS : PCL-diCOOH ratios, however, this offset was not sufficient, resulting in organic contents closer to 80 wt% than 75 wt%.

**Table 2:** SiO_2_–CaO/PTHF/PCL-diCOOH hybrids synthesised with different calcium sources with molar ratio of TEOS : Ca of 60 : 40 (60S40C_Ca_-CL), where Ca is the calcium source used with corresponding final inorganic : organic ratios in wt%, measured using thermogravimetry analysis

Hybrid name (60S40C_Ca_-CL, where Ca is the calcium source used)	I : O ratio (wt%)
60S40C_cCME_-CL	27.7 : 72.3
60S40C_CH_-CL	21.4 : 78.6
60S40C_CE_-CL	19.4 : 80.6
60S40C_CEE_-CL	24.2 : 75.8

The greatest discrepancy was for the 60S40C_CE_-CL hybrid, with organic content being 80.6 wt%, which agrees with the FTIR (Fig. 1b), wherein the carbonyl band had higher relative intensity. This is due to the additional THF in the system, which polymerises *in situ* to PTHF.^8^ The initial priority was to synthesise the hybrid inks suitable for printing with optimisation of the I : O ratio carried out later.

### Effect of calcium source on properties of monolithic hybrids

**Fig. 2:**
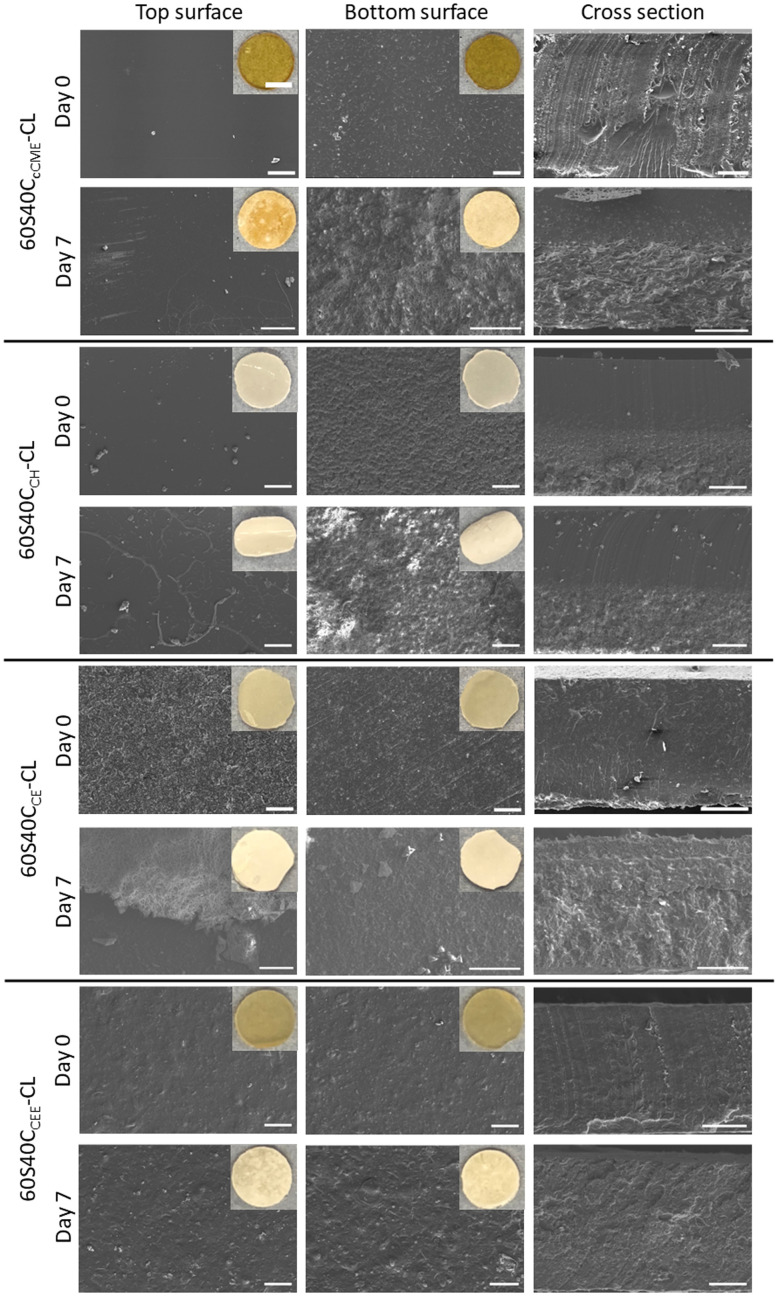
SEM micrographs of top surface, bottom surface, and cross-section, before and after 7 days immersed in SBF, of SiO_2_–CaO/PTHF/PCL-diCOOH hybrids with molar ratio of TEOS : Ca of 60 : 40, 60S40C_Ca_-CL, where Ca is the calcium source used (scale bar 200 μm). Insets show macro photographs of the top and bottom surfaces at the same timepoints (scale bar 2 mm). The top and bottom of the monoliths showed a difference in roughness due to the bottom being rougher because of their contact with the PTFE mould. The different roughness had an effect after the immersion in solution, with differences in deposit after 7 days in SBF, visible as well in the cross-section that shows the effect throughout the hybrid thickness.

**Fig. 3:**
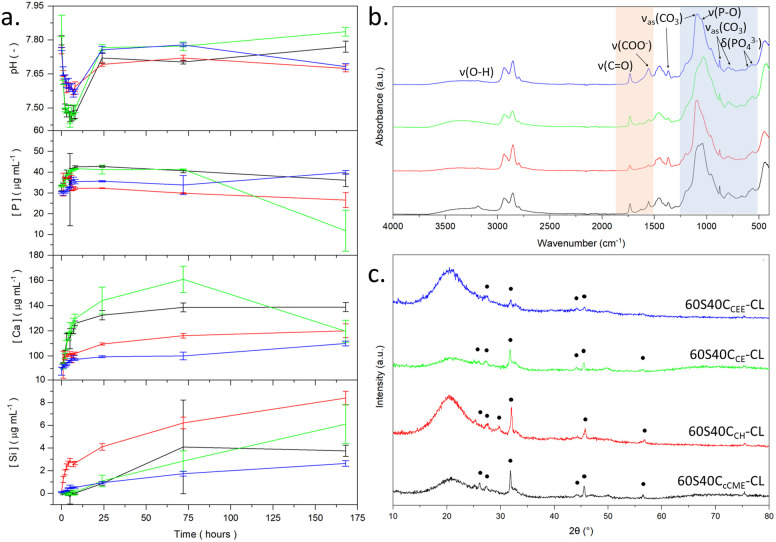
SBF studies of SiO_2_–CaO/PTHF/PCL-diCOOH hybrid monoliths with molar ratio of TEOS : Ca of 60 : 40, 60S40C_Ca_-CL, where Ca is the calcium source used, post 7 days immersion: (a) pH, phosphorous, calcium, and silicon, concentration measured *via* ICP-OES. (b) FTIR spectra after immersion; blue region highlights where the bands for calcium phosphate formation are, and the orange region shows bands for the interaction of calcium with the PCL-diCOOH. (c) XRD patterns with HCA peaks.^46^

The top of the hybrid monolith was defined as the surface in contact with air during drying, it appeared completely smooth for all compositions, whereas the bottom surface showed roughness from contacting the PTFE mould during the drying phase^45^ (Fig. 2). The release of calcium from monoliths synthesised with different calcium sources was investigated. After 7 days in SBF, a white deposit was visible to the naked eye on all hybrid compositions. For 60S40C_cCME_-CL and 60S40C_CH_-CL, more deposit was visible on the rougher bottom surface in micrographs (Fig. 2), likely to be due to the presence of increased nucleation sites. The 60S40C_CH_-CL monoliths had particles on the bottom surface before being immersed in SBF. The cross section confirms the presence of two phases, excess Ca(OH)_2_ could have deposited at the bottom of the mould during ageing and drying, as indicated by the XRD pattern (Fig. 1a). After 7 days in SBF, this seems to have accentuated: the surface with the deposited Ca(OH)_2_ showed more degradation in the cross section and a large amount of deposit. The 60S40C_CE_-CL and 60S40C_CEE_-CL monoliths appeared homogeneous through their cross-sections before and after 7 days immersion in SBF with similar deposit on the top and bottom surfaces, both having a rougher top surface than expected. From these observations, the 60S40C_CEE_-CL and 60S40C_CE_-CL hybrids were more promising for potential homogeneous degradation behaviour. The nature of the dissolution and precipitation was investigated by ICP, FTIR and XRD (Fig. 3).

Over 7 days in SBF, no burst release of calcium was observed for any hybrids, indicating that all calcium sources entered the hybrid network (Fig. 3a). Calcium release from 60S40C_cCME_-CL was consistent with the 60S40C_CME_-CL hybrid scaffolds investigated in previous work^28^ at ∼140 mg mL^−1^: 20 to 30 mg mL^−1^ higher than 60S40C_CEE_-CL and 60S40C_CH_-CL. 60S40C_CE_-CL had the highest calcium release, reaching 160 mg mL^−1^ after 72 h. The silica release was also controlled for all hybrid compositions, with the 60S40C_CH_-CL hybrid showing the highest concentration reaching 9 mg mL^−1^ after 7 days. This was similar to 60S40C_CME_-CL hybrids^27,28^ and other compositions reaching 2–6 mg mL^−1^ similar to 100S-CL calcium free hybrids.^27^ Such controlled release patterns confirms that the correct silica network with calcium incorporation was formed.

The 60S40C_CE_-CL was the only hybrid to show evidence of apatite formation from the ICP data, with the calcium and phosphate content dropping after 3 days. After 7 days in SBF, the FTIR spectra of all hybrid monoliths had the characteristic P–O bending bands at 550–600 cm^−1^ and P–O stretch at 1020 cm^−1^ indicative of orthophosphate, and 875 cm^−1^ from carbonate,^46^ CO_3_^2−^ (blue region, Fig. 3b). The carboxylate bands at 1550 cm^−1^ almost completely disappeared for all hybrid compositions, apart from 60S40C_CEE_-CL (orange region, Fig. 3b). After immersion, the 60S40C_CEE_-CL hybrid had a higher intensity carboxylate band than the carbonyl band, suggesting the slower calcium released of this hybrid likely originated from the calcium bonded to the silica network. In the XRD patterns (Fig. 3c), characteristic peaks of HCA were visible after 7 days of immersion for all hybrid compositions, at 27°, 32°, 34°, and 46° 2*θ*.^47^ The 60S40C_CEE_-CL hybrid showed the least number of peaks formed, as was expected from its slower Ca and silica dissolution (Fig. 3a), with less deposit and degradation visible on the monoliths (Fig. 2). This would not deter its use for bone repair, as the formation of HCA remains occurring, only slower, indicating a more sustained formation and the absence of calcium burst release. It can be hypothesised as a sign of calcium being better incorporated with the hybrid network.

The overall mass loss (Table 3) after 7 days in SBF, measured by weighing the samples before immersion and dried post-emersion, was difficult to quantify, as degradation of the hybrids occurs in parallel with formation of HCA at the hybrids' surface. It is impossible to separately obtain the mass loss from the degradation and the mass gained from the deposit. Although 60S40C_cCME_-CL did not seem to lose organic content with respect to inorganic in 7 days (Table 3), it did lose 18.4% of its original mass. This is an expected property in hybrids, where congruent degradation of the covalently linked organic and inorganic phases^9^ leads to a loss of overall mass but unchanging I : O ratios.^48^ The 60S40C_cCME_-CL hybrid showed an additional 12% mass loss compared to the 60S40C_CEE_-CL hybrid, which could indicate the silica network was modified to a greater extent by calcium in the 60S40C_cCME_-CL network, leading to faster degradation. This further validates the slower calcium and silica release rates of the 60S40C_CEE_-CL hybrid and slower formation of HCA (Fig. 3).

**Table 3:** Organic content of SiO_2_–CaO/PTHF/PCL-diCOOH hybrids (molar ratio of TEOS : Ca of 60 : 40, 60S40C_Ca_-CL, where Ca is the calcium source used) at day 0 and after immersion in SBF for 7 days. The delta organic content is the net difference observed between the two timepoints. The overall mass loss is also calculated between day 0 and 7

Hybrid monolith	Organic content day 0 (%)	Organic content day 7 (%)	Organic content delta (%)	Overall mass loss (%)
60S40C_cCME_	72.3	72.4	−0.1	18.4 ± 2.1
60S40C_CH_	78.6	74.8	3.8	8.8 ± 1.3
60S40C_CE_	80.6	63.3	17.3	13.7 ± 1.3
60S40C_CEE_	75.8	74.7	1.1	6.7 ± 1.5

After immersion, there was no visual degradation. All hybrids showed <4% difference in organic wt% before and after immersion, apart from the 60S40C_CE_-CL monolith, for which 17.3 wt% difference in organic content was measured, Table 3. This put in question the integrity of this hybrid network, potentially overly disrupted from the calcium incorporation. From chemical analysis, the calcium powder sources were the least reliable to synthesise hybrids with, but printability and mechanical properties had to be assessed.

### Direct ink writing hybrid scaffolds with different calcium sources

**Fig. 4:**
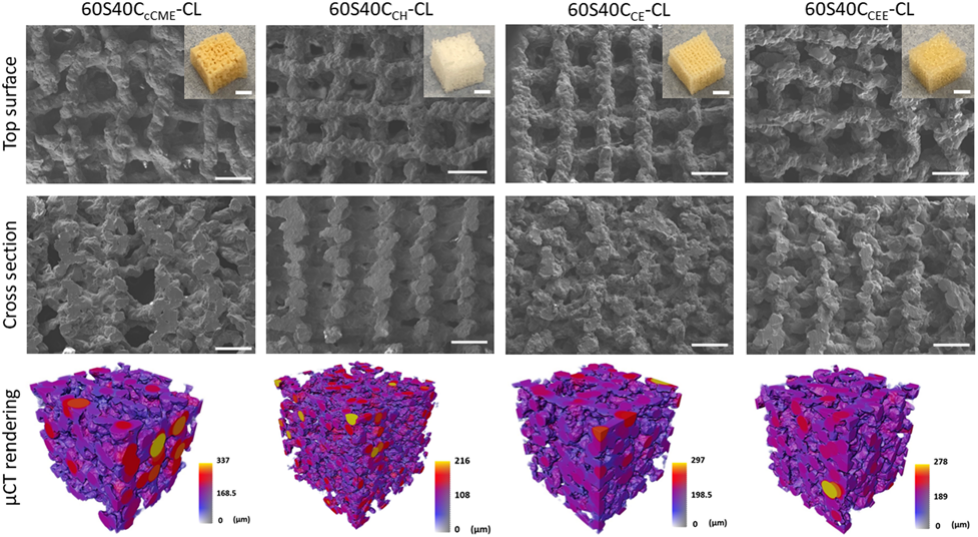
SEM micrographs and photographs (insets) of 3D printed SiO_2_–CaO/PTHF/PCL-diCOOH hybrids with molar ratio of TEOS : Ca of 60 : 40, 60S40C_Ca_-CL, where Ca is the calcium source used; μCT imaging and analysis of a volume of interest (2600 × 2600 × 2600 μm) showing the 3D rendering of the scaffold strut structure, coloured by their diameter size in μm.

Having assessed the capability of all 60S40C_Ca_-CL hybrids to form HCA with no notable pH change, their printability and the mechanical properties of printed scaffolds were assessed to define the best calcium source for making bone implants. “Printability” refers to the printing window, which is the duration of time for which the ink has suitable viscosity for printing (extrusion) in the form of a scaffold. A low viscosity results in collapse. Excess gelling from ongoing polymerisation leads to struts not bonding to each other properly, as polymerisation can become too advanced before printing. The first observation after printing all hybrid compositions was that when the calcium source was in powder form (CH and CE), the correct printing gelation was attained almost immediately after defrosting the inks from −80 °C, however these hybrid inks also had a shorter printing window (<45 min compared to 1–2 h). This made it difficult to print multiple scaffolds without the ink excess gelling, leading to unreliable scaffold structures and material waste. As for liquid Ca sources, CEE was much easier to print, with a gelation time of 2 h, faster than CME and cCME (4 h), and use of CEE produced a long printing window (1–2 h) due to shear thinning properties, enabling the printing of large structures. All calcium sources were able to be printed into scaffolds with a 0.20 nozzle size, a strut separation of 1 mm, and cut to 5 × 5 × 5 mm once dried (Fig. 4).

The pore sizes of the 60S40C_CE_-CL and 60S40C_CH_-CL scaffold hybrids showed an additional shrinkage of 11 and 13%, respectively, compared to the 60S40C_cCME_-CL and 60S40C_CEE_-CL scaffold structures (Table 4). Overall, the 60S40C_CEE_-CL hybrid sol was the easiest to print, with a reliable gelation, and ongoing polymerisation for printed struts to bond correctly. Although 60S40C_cCME_-CL measured to a similar final structure, the ink was more difficult to print, and struts did not bond as well due to a longer gelation window, resulting in an unreliable scaffold (Fig. 4).

**Table 4:** Strut and pore sizes of 3D printed SiO_2_–CaO/PTHF/PCL-diCOOH hybrids with a 0.20 mm nozzle and molar ratio of TEOS : Ca of 60 : 40, 60S40C_Ca_-CL, where Ca is the calcium source used, comparing measurement taken using μCT data and SEM micrographs *via* ImageJ

	μCT measurements			SEM ImageJ measurements		
	Strut size (μm)	Channel size (μm)	Porosity (%)	Horizontal channels (μm)	Vertical channels (μm)	Strut size (μm)
60S40C_cCME_-CL	253 ± 117	166 ± 54	51 ± 3	451 ± 68	114 ± 35	168 ± 19
60S40C_CH_-CL	97 ± 36	217 ± 110	61 ± 1	391 ± 65	112 ± 33	148 ± 17
60S40C_CE_-CL	221 ± 107	143 ± 38	57 ± 3	399 ± 60	121 ± 24	168 ± 15
60S40C_CEE_-CL	256 ± 114	137 ± 38	60 ± 0.7	452 ± 79	115 ± 27	154 ± 20

The porosity of these hybrid scaffolds ranges from 51% to 61%, which does not match trabecular bone (70 to 95%),^49^ however, the aim here was to match the scaffold's mechanical properties to bone while having an open interconnectivity to allow for tissue ingrowth, vascularisation, and nutrient delivery. All scaffolds had open interconnected channels according to μCT images, generating an open network for bone tissue and vascular ingrowth. Fig. 4 shows the strut size of hybrid scaffolds *via* 3D colour maps with μCT measurements. For 60S40C_cCME_-CL, 60S40C_CE_-CL, and 60S40C_CEE_-CL, μCT measurements show larger strut size compared to SEM ImageJ measurements. However, 60S40C_CH_-CL had the lowest strut size in μCT measurements due to large shrinkage. It is important to note that μCT measurements account for 3D geometry as well as intersections, while SEM is limited to the 2D plane (defined as vertical, measuring the gap between struts in the *z* plane, and horizontal, in the *x*–*y* plane, channels). This explains the discrepancy between the two measuring methods. Nonetheless, both approaches showed that all scaffolds achieved an open interconnective channel network, with a size range of 400–500 μm, ideal for bone regeneration.^50^

**Fig. 5:**
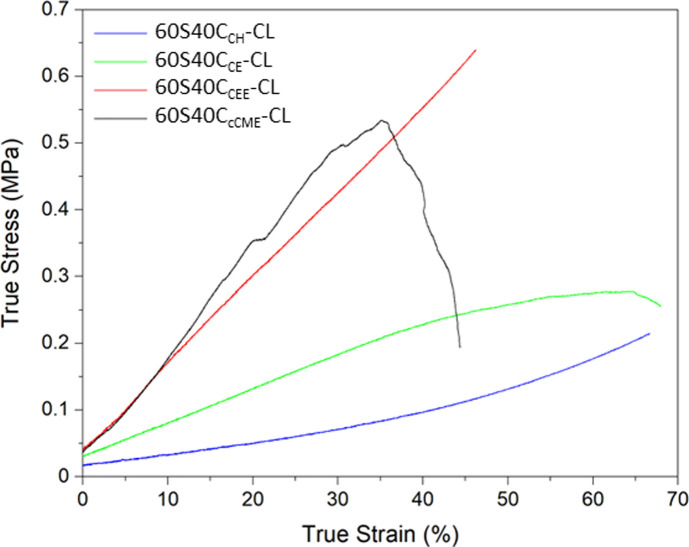
Typical stress–strain curves of the 3D printed SiO_2_–CaO/PTHF/PCL-diCOOH hybrids scaffolds with molar ratio of TEOS : Ca of 60 : 40, 60S40C_Ca_-CL, where Ca is the calcium source used.

Mechanical properties of the scaffolds are crucial as bone implants need to match the host tissue to allow mechanical functionality, prevent stress shielding and promote biomechanical signals for osteogenesis. Here, the calcium source showing the most appropriate mechanical properties when used to synthesise a hybrid was identified. The hybrid scaffolds were compressed to failure, showing different mechanical properties with some exhibiting a yield stress point, others showing a *J*-curve trend (Fig. 5). From these results, a safe strain range, where the hybrid behaviour was considered elastic, was identified for dynamic mechanical analysis to evaluate the storage modulus of the hybrids.

The 60S40C_cCME_-CL and 60S40C_CE_-CL hybrid scaffolds showed a linear elastic region, with a yield point and an ultimate strength point, after which failure occurred more rapidly. The 60S40C_cCME_-CL hybrid showed some steps, indicative of some struts failing prematurely, likely at the edges of the scaffolds where stress can concentrate. The 60S40C_CE_-CL did not show as distinct a yield point between its elastic and plastic regions. The yield point is visible at 40% by a slight change in gradient and plateauing of the curve. On the other hand, the 60S40C_CEE_-CL and 60S40C_CH_-CL hybrid scaffolds showed *J*-curve behaviours, their yield point also corresponding to their failure point, with no sudden failure observed during the tests. Loading of *J*-curves is elastic and reversible, with unloading occurring on the same curve.^51^ The 60S40C_CEE_-CL hybrid reached the highest average stress of 0.55 MPa at a strain of approximately 53% (Table 5). Overall, calcium addition increased the strain to failure reached, regardless of the calcium source used.^27^

**Table 5:** Mechanical properties of the 3D printed SiO_2_–CaO/PTHF/PCL-diCOOH hybrids with molar ratio of TEOS : Ca of 60 : 40, 60S40C_Ca_-CL, where Ca is the calcium source used hybrid scaffolds, with 100S-CL^27^ as control

	True stress (MPa)	True strain (%)	Toughness modulus (MPa)	Storage modulus – 1 Hz – strain 5–9% (MPa)
60S40C_cCME_-CL	0.38 ± 0.15	44.9 ± 14.6	0.11 ± 0.06	2.14 ± 0.14
60S40C_CH_-CL	0.20 ± 0.01	66.5 ± 10.8	0.05 ± 0.01	0.74 ± 0.07
60S40C_CE_-CL	0.28 ± 0.03	58.7 ± 10.5	0.11 ± 0.01	0.97 ± 0.04
60S40C_CEE_-CL	0.55 ± 0.08	53.1 ± 9.8	0.13 ± 0.03	2.26 ± 0.14
100S-CL^28^	0.36 ± 0.14	31.3 ± 9.5	0.06 ± 0.01	—

The toughness modulus confirmed 60S40C_CEE_-CL hybrids had the highest toughness. The storage modulus was measured between 5 and 9% strain, within the elastic region of all the hybrid scaffolds, as no plastic deformation occurred. The 60S40C_cCME_-CL and 60S40C_CEE_-CL scaffolds had very similar moduli, over double that of the other compositions. None reached values matching trabecular bone,^49^ however, due to the hybrid's tailorable properties, optimisation is possible by altering the final I : O ratio and the printing parameters. The trend showing an increase in toughness with the addition of calcium is thought to be due to calcium cations forming stabilising complexes with lone pairs on the ester linkages along the PCL backbone, and with any remaining unreacted carboxylate terminal groups. The carbonyl oxygen has two lone electron pairs and slight excess of negative charge to form a stabilising complex with calcium, oxygen acting as a ligand in the metal complex.^28,52^ Only 60S40C_CH_-CL showed a lower toughness modulus, it was hypothesised that polymerisation was complete before printing, giving very poor mechanical structure to the hybrid. This hybrid scaffold showed a longer period of elastic deformation, with a true strain to failure reaching >66% on average.

The CEE calcium source was chosen as it showed an increase in printability, mechanical properties, and congruent degradation with sustained calcium and silica release, with a regular interconnected channel network.

### Optimisation of the 70S30C_CEE_-CL hybrid for bone implant

**Fig. 6:**
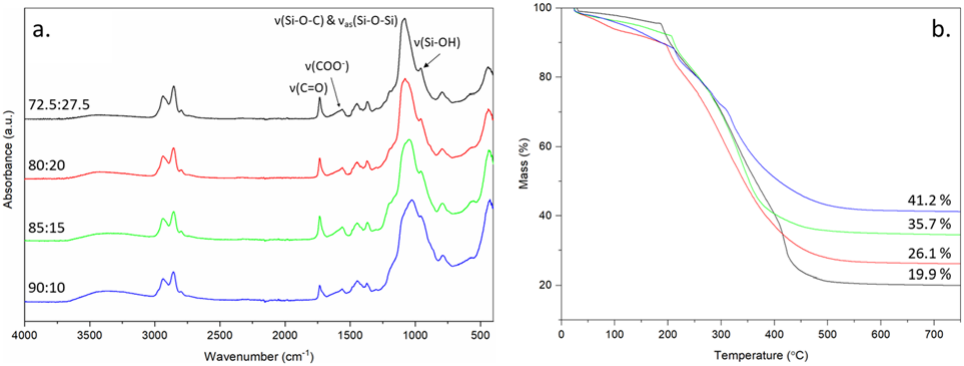
FTIR and TGA characterisation of 70S30C_CEE_-CL hybrid scaffolds made with TEOS : CEE molar ratio of 70 : 30 and TEOS : PCL-diCOOH weight ratios of: 72.5 : 27.5, 80 : 20, 85 : 15, and 90 : 10, (a) FTIR, (b) TGA traces showing the final value corresponding to the inorganic content of each hybrid composition.

From previous work, a TEOS : calcium molar ratio of 70 : 30 was highlighted as optimal for osteogenic properties in 70S30C_CME_-CL hybrids^28^ and therefore the same ratio was used here to produce bone scaffolds with CEE as the calcium source. The focus was on optimising the mechanical properties of the 70S30C_CEE_-CL hybrid by altering the scaffold strut size whilst keeping a 400–500 μm channel size and altering the TEOS : PCL-diCOOH weight ratio from 72.5 : 27.5 to 90 : 10 wt% (Fig. 6). By increasing the inorganic content, the hybrid strength should increase, paired with a reduction in elasticity.

**Fig. 7:**
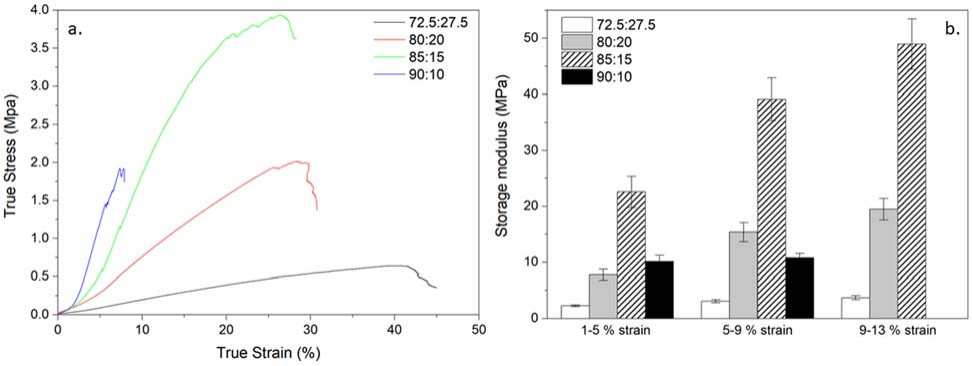
Stress strain curves and storage modulus values of 70S30C_CEE_-CL hybrid scaffolds, printed with a 0.40 mm nozzle size, synthesised with a 70 : 30 TEOS : CEE molar ratio and TEOS : PCL-diCOOH weight ratios of: 72.5 : 27.5, 80 : 20, 85 : 15, and 90 : 10. Shown for all compositions: (a) typical stress strain curve, and (b) the average storage modulus (from DMA) obtained at strain ranges of 1–5%, 5–9%, and 9–13% (the 90 : 10 wt% ratio being too brittle to be measured at the 9–13% range).

All hybrid compositions formed the silica network, with relative intensity of the carbonyl stretching band at 1730 cm^−1^ reducing, with respect to Si–O stretch, as the amount of TEOS increased (Fig. 6). The inorganic content was also confirmed by TGA (Fig. 6b), increasing from 19.9% to 41.2% as TEOS : PCL-diCOOH weight ratios were increased from 72.5 : 27.5 to 90 : 10. The hybrids were tested under compression and DMA (Table 6 and Fig. 7) to investigate the effect of altering the TEOS : PCL-diCOOH weight ratio and strut size of the scaffolds. For scaffolds with TEOS : PCL-diCOOH ratio of 72.5 : 27.5 wt%, changing the nozzle size between the 0.20 and 0.25 mm nozzle sizes had little effect but the yield stress reduced when the larger 0.40 mm nozzle was used. Scaffolds with a TEOS : PCL-diCOOH ratio of 80 : 20 wt% showed a reduction in both the yield stress and strain when printed with the 0.25 mm nozzle compared to the 0.20 mm, but the yield stress increased with the 0.40 mm nozzle, reaching 1.99 ± 0.35 MPa true yield stress and 26.8 ± 2.4% true yield strain. The strain being greater than 20% is above the requirement to match trabecular bone.^53^ Increasing the inorganic content should enable further increase in yield stress reached whilst keeping the strain within the range of trabecular bone.

**Table 6:** Mechanical properties of the 70S30C_CEE_-CL hybrid scaffolds printed with varying nozzle sizes and TEOS : PCL-diCOOH wt% ratios

TEOS : PCL-diCOOH (wt%)	I : O ratio (wt%)	Nozzle size (mm)	True yield stress (MPa)	True yield strain (%)	Toughness modulus (MPa)	Storage modulus – 1 Hz – strain 5–9% (MPa)
72.5 : 27.5		0.20	1.01 ± 0.07	49.0 ± 6.0	0.28 ± 0.05	2.92 ± 0.21
	19.9 : 80.1	0.25	0.93 ± 0.15	41.0 ± 5.8	0.24 ± 0.05	2.73 ± 0.27
		0.40	0.51 ± 0.10	40.7 ± 2.7	0.15 ± 0.01	3.08 ± 0.28
80 : 20		0.20	0.93 ± 0.07	32.3 ± 2.1	0.25 ± 0.03	9.50 ± 0.67
	26.1 : 73.9	0.25	0.61 ± 0.20	22.5 ± 4.5	0.11 ± 0.04	8.42 ± 0.26
		0.40	1.99 ± 0.35	26.8 ± 2.4	0.32 ± 0.06	15.38 ± 1.73
85 : 15		0.20	0.62 ± 0.02	11.05 ± 1.7	0.05 ± 0.01	10.55 ± 0.60
	35.7 : 64.3	0.25	0.54 ± 0.09	10.9 ± 0.8	0.04 ± 0.01	31.13 ± 2.93
		0.40	3.15 ± 0.54	25.9 ± 3.9	0.58 ± 0.06	39.10 ± 3.81
90 : 10		0.20	1.37 ± 0.23	7.5 ± 1.4	0.13 ± 0.02	—
	41.2 : 58.8	0.25	2.57 ± 0.51	9.64 ± 2.7	0.09 ± 0.06	—
		0.40	1.45 ± 1.00	12.4 ± 3.8	0.12 ± 0.08	10.81 ± 0.76

Scaffolds with TEOS : PCL-diCOOH ratio of 85 : 15 wt% behaved in a similar way to the scaffolds with TEOS : PCL-diCOOH ratio of 80 : 20 wt%, showing an even more significant increase effect of strut size on true yield stress, reaching 3.15 ± 0.54 MPa for the 0.40 mm nozzle size, with corresponding strain still reaching 25 ± 3.9%. This yield stress reached the lower end of the 2–12 MPa range for trabecular bone.^53^ Its storage modulus steadily increased in all strain ranges investigated, similarly to the 80 : 20 wt% hybrid but reaching over double this composition's values, and close to 50 MPa in the 9–13% strain range (Fig. 7b). When scaffolds with TEOS : PCL-diCOOH ratio was increased to 90 : 10 wt%, strain and stress to failure decreased to 12% and 1.45 MPa, respectively (0.40 mm nozzle), even though a high strength of 2.57 ± 0.51 MPa at 10% strain was reached when printed with a 0.25 mm nozzle, indicating brittleness. The low strain and brittle failures observed during compression to failure and the strain range variabilities made it impossible to correctly measure the storage modulus of this composition in the 9–13% range. For other compositions, the storage modulus mirrored the behaviour of the yield stress, with a large increase for the 85 : 15 wt% composition, reaching a mean of 39 MPa for the 0.40 mm nozzle size (Table 6). This is still more than an order of magnitude lower than bone due to the increased elasticity of the hybrid: the elastic compressive modulus of trabecular bone established to range between 0.8–2.7 GPa.^53,54^ Current metallic implants show a larger stiffness, leading to stress shielding and they are non-biodegradable. Titanium implants made of Ti_6_Al_4_V with 700 μm pores and 300 μm struts size have elastic modulus of 110 GPa and yield strength between 880–900 MPa.^55^ It is difficult to find mechanical properties of current synthetic bone implants for comparison, however, values for the stiffness of materials often used can be found. Ceramic implants such as tricalcium phosphate and hydroxyapatite are 60–75 GPa and 80–110 GPa, respectively^2^ (specific scaffold dimensions not specified). Ceramics are known to be brittle and show no cyclic loading abilities. Polyetheretherketone (PEEK) and hydroxyapatite composites showed compressive strength and modulus of 36.45 MPa and 2.71 GPa, respectively,^2^ seemingly matching bone values better than previously mentioned materials, however, they are non-degradable. Hybrids solve the issues faced by composites and removed the risk of stress shielding from titanium implants. Scaffolds with a TEOS : PCL-diCOOH ratio of 85 : 15 wt% was the most promising and reproducible hybrid composition and was chosen as the composition to further evaluate for its use for bone implants, named 70S30C_CEE_-85S15CL.

**Fig. 8:**
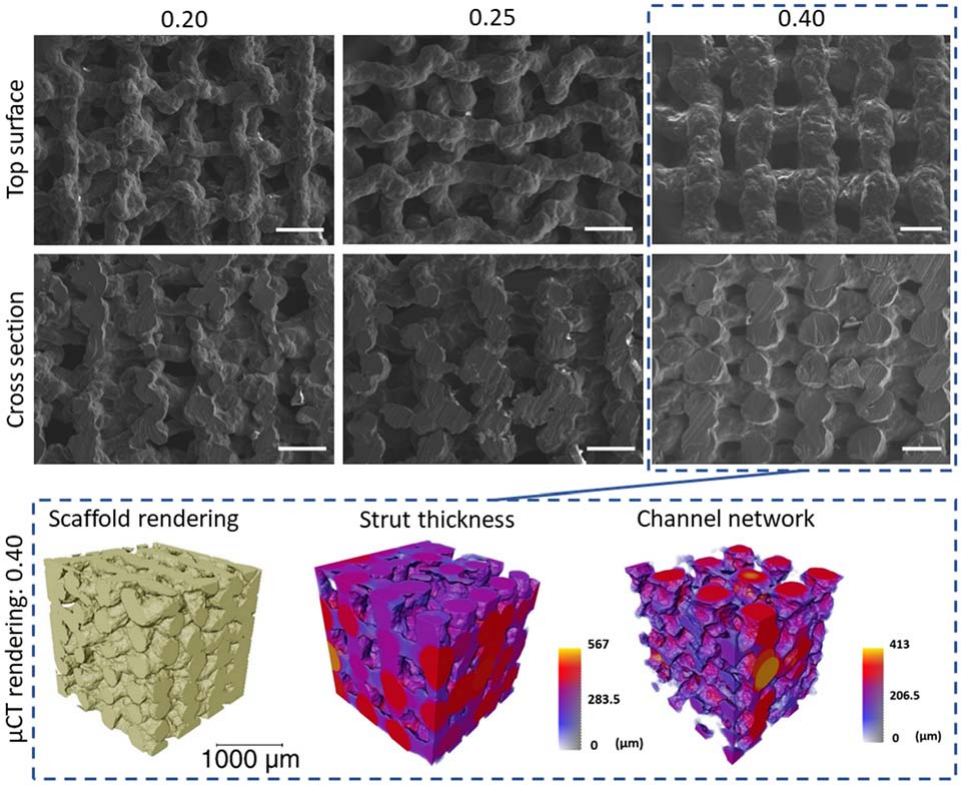
SEM micrographs of the top surface and cross sections of 70S30C_cCME_-85S15CL hybrid scaffolds 3D printed *via* Direct Ink Writing with nozzle size 0.20, 0.25, and 0.40 mm, scale bar 500 μm. μCT imaging and analysis of a volume of interest (2600 × 2600 × 2600 μm) of the 0.40 mm nozzle size hybrid scaffold showing the 3D rendering of the scaffold, the strut thickness coloured by their diameter size and the 3D rendering of the interconnected channel network (negative of the strut rendering), with size scale in μm.

The different final strut sizes for 70S30C_CEE_-85S15CL scaffolds are observable in Fig. 8. The SEM micrographs show the top surface and cross section of the scaffolds printed with different nozzle sizes (0.20, 0.25, and 0.40 mm) after ageing and drying. This enabled the measurement of the pore size horizontally and vertically, as well as the strut size.

The 0.40 mm nozzle size 70S30C_CEE_-85S15CL scaffold showed a regular 3D structure, with a μCT measured strut size of 297.9 ± 78.1 μm and a porosity of 38.9 ± 0.7%, due to the increased strut size whilst keeping the printed pores within the desired range for bone regeneration 350–450 μm^50^ (Table 7). With open interconnectivity of the channels confirmed *via* μCT, visible in the strut negative rendering (Fig. 8).

**Table 7:** The horizontal and vertical pores, and strut size measured on the SEM micrographs for nozzle sizes of 0.20, 0.25, and 0.40 mm and μCT for nozzle sizes of 0.20 and 0.40 mm

Nozzle size (mm)	SEM ImageJ measurements			μCT measurements		
	Horizontal channel size (μm)	Vertical channel size (μm)	Strut size (μm)	Porosity (%)	Strut size (μm)	Channel size (μm)
0.20	439 ± 69	88 ± 23	168 ± 17	59.6 ± 0.7	137.1 ± 31.7	255.4 ± 114.4
0.25	425 ± 68	125 ± 30	234 ± 33	—	—	—
0.40	402 ± 52	176 ± 39	390 ± 36	38.9 ± 0.7	297.9 ± 78.1	240.5 ± 89.6

The channel size was measured to be 240.5 ± 98.6 μm *via* μCT, corresponding to the average channel size in all spatial directions; it was not possible to differentiate between vertical or horizontal channel sizes as defined for SEM measurements, 176 ± 39 μm and 402 ± 52 μm, respectively.

All scaffolds, regardless of nozzle size (0.20, 0.25, and 0.40 mm) showed a regular structure and predictable final pore and strut sizes (Fig. 8 and Table 7), confirming the reproducibility and reliability of the 70S30C_CEE_-85S15CL hybrid ink. The final mechanical properties of the 0.40 mm nozzle hybrid scaffold were the only ones within the trabecular bone properties, Table 6, required for a bone regenerating implant to share load with the host tissue, as well as its open interconnected network to allow for tissue ingrowth and vascularisation.

### Metabolic activity of human bone mesenchymal stem cells with 70S30C_CEE_-85S15CL conditioned media

**Fig. 9:**
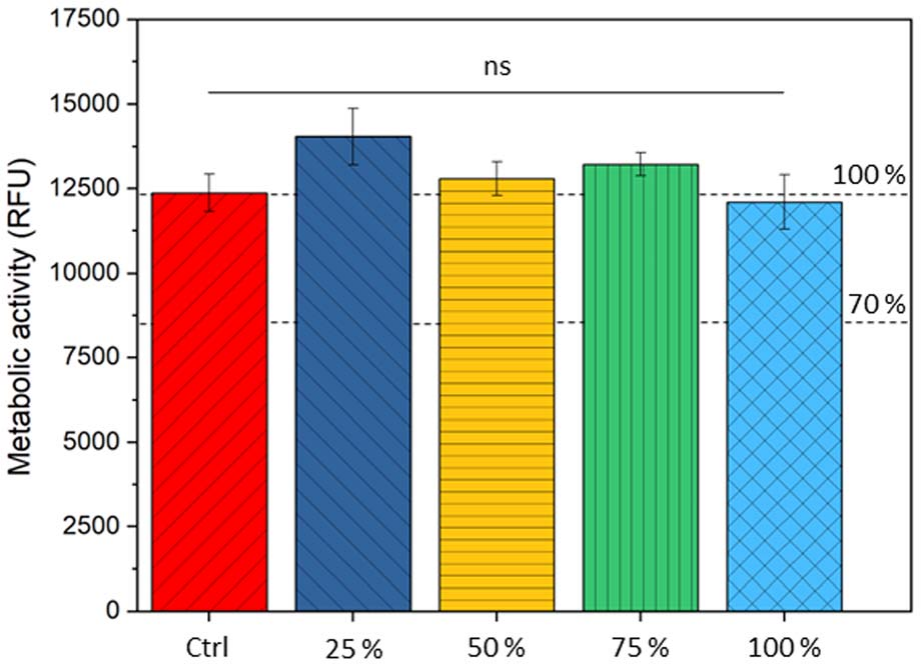
Effect of 70S30C_CEE_-85S15CL hybrid dissolution products (and diluted to 25, 50, 75%) on the metabolic activity of hBMSCs. No significant differences in metabolic ability were observed.

The viability of hBMSCs was assessed when cultured with dissolution products of optimised 70S30C_CEE_-85S15CL. Metabolic activity was assessed in samples prior to (control MSCM) and after 24 h exposure to serial dilution of ionic release from the optimised hybrid (25, 50, 75, and 100% of conditioned media), Fig. 9.

Non-significant differences in metabolic activity were observed with dissolution products of 70S30C_CEE_-85S15CL hybrids. The 70S30C_CME_-CL evaluated in a previous study showed similar response for cells exposed to conditioned media, where a non-significant decrease of 12% for the 100% conditioned media was observed.^28^ The silicon and calcium concentration for this hybrid's conditioned media was of 70.0 ± 3.1 μg mL^−1^ and 187.3 ± 12.5 μg mL^−1^, respectively.^28^ Media conditioned with optimised 70S30C_CEE_-85S15CL hybrid had a silicon and calcium concentration of 13.5 ± 0.2 μg mL^−1^ and 143.9 ± 2.7 μg mL^−1^, respectively. The slower release of silica could explain the lower drop in cell viability for the 70S30C_CEE_-85S15CL hybrid and indicate a better silica network formation for this optimised hybrid composition.

## Conclusions

Calcium ethoxyethoxide was found to be the optimal calcium source for synthesis of SiO_2_–CaO/PTHF/PCL-diCOOH hybrid scaffolds, compared to cCME, CH, and CE. Although all sources enabled calcium incorporation into the hybrid network at room temperature, hybrids made with CEE showed the best printability, whilst having a controlled biodegradability with a sustained release of calcium and silica species, forming hydroxycarbonate apatite within 7 days of immersion in SBF. By changing the TEOS : CEE molar ratio to 70 : 30, increasing the inorganic content to 35.7% (TEOS : PCL-diCOOH ratio of 85 : 15 wt%) and the strut thickness to 300 μm (with interconnected channel size of >400 μm and porosity of 38.9%), it resulted in the highest final mechanical properties. The stress at failure was 3.13 ± 0.54 MPa, and toughness modulus 0.58 ± 0.06 MPa, for a corresponding strain of 26%. The strength of the optimised 70S30C_CEE_-85S15CL hybrid scaffold was within the trabecular bone range of 2–12 MPa,^53^ with an open porosity of >38%, and did not show cytotoxicity when hBMSCs were exposed to its conditioned media. This is the first bone implant which can recover from deformation within the trabecular bone range and an open network, whilst degrading to form the precursor to osteogenesis. This confirms the 70S30C_CEE_-85S15CL hybrid implant shows promise for bone implants.

## Data availability

Data for this article, including mechanical, thermal, chemical and cell viability raw data, measurements, and micrographs are available at Zenodo at https://doi.org/10.5281/zenodo.13838937.

## Author contributions

AH, FT, SC, and JJ developed experimental protocols and designed the experiments. AH produced all the samples used in the experiment, conducted experiments, analysed, and interpreted the data. SC analysed and interpreted data. JL and PL conducted the μCT imaging experiments, analysed, and interpreted the data. AH, JT, JC, JL, JJ, and GJ developed and designed experimental *in vitro* protocols. JT performed the *in vitro* experiments. JJ, PL, and GL obtained funding and supervised the project. AH and JJ wrote the manuscript, all authors reviewed it.

## Conflicts of interest

There are no conflicts to declare.
